# Supplementary figures and images for: Effective asexual reproduction of a widespread soft coral: comparative assessment of four different fragmentation methods
Source: PeerJ. 2022 Jan 19;10:e12589. doi: 10.7717/peerj.12589 (PMC8783554; doi:10.7717/peerj.12589)

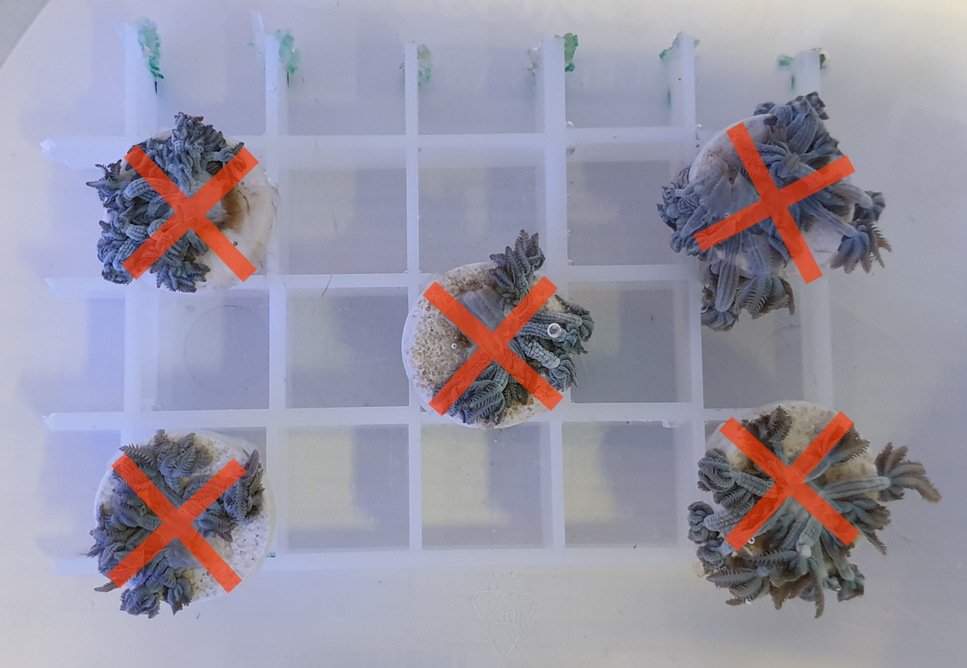

Supplement: Supplemental Information 2 — Rubber bands are marked with red highlights for better observation since the rubber bands that were originally used were transparent. [file peerj-10-12589-s002.png]

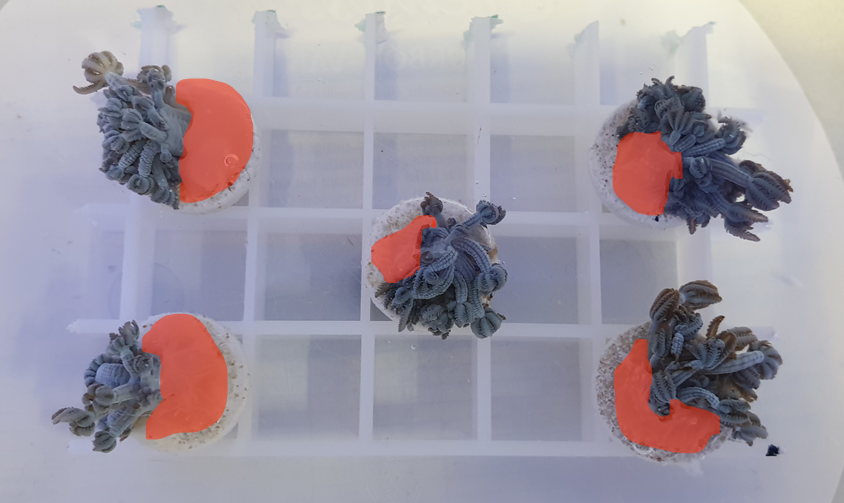

Supplement: Supplemental Information 3 — The glue areas are colored in red for better observation. [file peerj-10-12589-s003.png]
